# Biphasic bone substitutes coated with PLGA incorporating therapeutic ions Sr^2+^ and Mg^2+^: cytotoxicity cascade and *in vivo* response of immune and bone regeneration

**DOI:** 10.3389/fbioe.2024.1408702

**Published:** 2024-06-24

**Authors:** Yanru Ren, Ole Jung, Milijana Batinic, Kim Burckhardt, Oliver Görke, Said Alkildani, Alexander Köwitsch, Stevo Najman, Sanja Stojanovic, Luo Liu, Ina Prade, Mike Barbeck

**Affiliations:** ^1^ Clinic and Policlinic for Dermatology and Venereology, University Medical Center Rostock, Rostock, Germany; ^2^ Institute of Materials Science and Technology, Chair of Advanced Ceramic Materials, Technical University Berlin, Berlin, Germany; ^3^ BerlinAnalytix GmbH, Berlin, Germany; ^4^ Biotrics Bioimplants AG, Berlin, Germany; ^5^ Department of Biology and Human Genetics, Faculty of Medicine, University of Niš, Niš, Serbia; ^6^ Scientific Research Center for Biomedicine, Department for Cell and Tissue Engineering, Faculty of Medicine, University of Niš, Niš, Serbia; ^7^ College of Life Science and Technology, Beijing University of Chemical Technology, Beijing, China; ^8^ FILK Freiberg Institute, Freiberg, Germany

**Keywords:** bioactive ions, magnesium, strontium, biphasic bone substitute, biocompatibility, bone remodeling, immune response, *in vivo*

## Abstract

The incorporation of bioactive ions into biomaterials has gained significant attention as a strategy to enhance bone tissue regeneration on the molecular level. However, little knowledge exists about the effects of the addition of these ions on the immune response and especially on the most important cellular regulators, the macrophages. Thus, this study aimed to investigate the *in vitro* cytocompatibility and *in vivo* regulation of bone remodeling and material-related immune responses of a biphasic bone substitute (BBS) coated with metal ions (Sr^2+^/Mg^2+^) and PLGA, using the pure BBS as control group. Initially, two cytocompatible modified material variants were identified according to the *in vitro* results obtained following the DIN EN ISO 10993-5 protocol. The surface structure and ion release of both materials were characterized using SEM-EDX and ICP-OES. The materials were then implanted into Wistar rats for 10, 30, and 90 days using a cranial defect model. Histopathological and histomorphometrical analyses were applied to evaluate material degradation, bone regeneration, osteoconductivity, and immune response. The findings revealed that in all study groups comparable new bone formation were found. However, during the early implantation period, the BBS_Sr^2+^ group exhibited significantly faster regeneration compared to the other two groups. Additionally, all materials induced comparable tissue and immune responses involving high numbers of both pro-inflammatory macrophages and multinucleated giant cells (MNGCs). In conclusion, this study delved into the repercussions of therapeutic ion doping on bone regeneration patterns and inflammatory responses, offering insights for the advancement of a new generation of biphasic calcium phosphate materials with potential clinical applicability.

## 1 Introduction

Although bone tissue possesses the capacity to repair and regenerate after injury or surgical treatment, the natural healing can be a slow process that often fails to restore the original structure and functionality of bone (Giannoudis et al., 2005; Ansari, 2019). Large maxillofacial bone defects, resulting from trauma injuries, cancer treatments, or tooth loss, frequently necessitate the application of bone grafting procedures. (Weiss et al., 1999; Karhuketo et al., 2022). Autologous bone transplants continue to be considered as the most effective substitute of choice for bone regeneration (Albrektsson and Johansson, 2001; Karhuketo et al., 2022). However, a limited amount of autologous bone and the donor site morbidity are factors that limit its application, which has been promoting the development of bone substitute materials (BSMs) within the last decades (Henkel et al., 2013). Synthetic BSMs, particularly calcium phosphates like hydroxyapatite (HA) and beta tri-calcium phosphate (β-TCP) ceramics, are widely used due to their biocompatibility and resemblance to natural bone (LeGeros, 2008; Bellucci et al., 2016). Hydroxyapatite (HA), a key component of teeth and bone, boasts impressive mechanical strength but degrades at a slower rate, which is usually maintained at least several years after implantation, partially hindering bone regeneration up to a complete *restitutio ad integrum* (Bellucci et al., 2016; Fernandez de Grado et al., 2018). ß-TCP is also a biocompatible and bioresorbable material, which can be completely degraded in approximately 13–20 weeks after implantation and is then often replaced by remodeled new bone but its fast degradation profile can lead to decreased osteoconductive properties (Chazono et al., 2004). Based on their relatively opposite resorption behaviors, HA is often combined with ß-TCP in form of biphasic bone substitutes (BBS). The mixture of HA and ß-TCP combines the advantages even in view of the cellular degradability and solubility and avoids the disadvantages of these two components (Ghanaati et al., 2012). Although, BBS present biocompatibility with improved osteoconductivity and bone regeneration, there still remains significant room for improvement of synthetic BSMs to reach regenerative capacities of the “golden standard” autologous bone transplants, especially in view of osteogenesis, osteoinduction, and vascularization (Fernandez-Bances et al., 2013; Jeong et al., 2019).

In response, increasing attention has been shifted to BSM with bioactive cations, such as magnesium (Mg^2+^), zinc (Zn^2+^), copper (Cu^2+^), strontium (Sr^2+^), and others. In particular, Mg^2+^ and Sr^2+^ in wide concentration ranges do not generate distinct cytotoxicity (Bracci et al., 2009). Sr^2+^ is beneficial in improving osteoblast proliferation, and hence promoting bone formation by increased matrix synthesis through the calcium-sensing receptor and the Extracellular Signal-Regulated Kinase (ERK) signaling pathway (Li et al., 2020; Chen et al., 2022). In addition, it has been confirmed, that Mg^2+^, known as the fourth most abundant element in the human body, has shown to provide great advantages as a bioactive substance in biomaterials for repairing bone defects (Hung et al., 2019; Ballouze et al., 2021). The released Mg^2+^ has proved to promote new bone formation and to increase the expression of osteogenic markers, and thus enhance osteogenesis and osseointegration (Ballouze et al., 2021; Fan et al., 2022). Furthermore, adaptions of BSMs even in view of the material-induced immune response, especially on pro- and anti-inflammatory cells such as macrophages but also multinucleated giant cells (MNGCs), and the combined immunomodulatory properties have been in the focus of biomaterials research over the last decade (Zheng et al., 2018; Yang et al., 2022; Zhao et al., 2022). In this context, bioactive cations have shown to be capable of regulating the material-associated inflammatory process, which is another important regenerative factor for bone healing (Rolvien et al., 2018; Abels et al., 2021; Barbeck et al., 2021; Barbeck et al., 2022; Alkildani et al., 2023). Hence, exploring the inflammatory response provoked by bone substitute materials containing metal ions, and their impact on modulating bone regeneration *in vivo*, is a matter of significant interest.

Moreover, polymer coatings also have been cited as an effective technique for modifying the bio-functionality of tissue engineering materials (Maadani et al., 2023). Poly-lactic-co-glycolic acid (PLGA), a group of FDA-approved biodegradable polymers, holds great promise for drug encapsulation, biomaterial degradation modulation and mechanical properties modulation owing to its exceptional physical strength and high biocompatibility (Klose et al., 2008). In the present study, 18 types of surface modifications with metal ions (Sr^2+^ and/or Mg^2+^) and PLGA on basis of an already approved and biocompatible BBS (maxresorb, botiss biomaterials GmbH, Zossen, Germany) were prepared. The pure BBS was applied as a control group in this study (Konermann et al., 2014; Trajkovski et al., 2018; botiss biomaterials Gmb, 2024). It was hypothesized that the addition of the bioactive divalent cations Sr^2+^ and/or Mg^2+^ will affect the inflammatory tissue reaction to the biomaterials as well as improve bone tissue regeneration, leading to more satisfactory bone repair outcomes. To evaluate these hypotheses, an *in vitro* cytotoxicity study with the above mentioned 30 modified BBS materials was initially conducted according to the DIN ISO 10993-5 protocol. Based on the *in vitro* results the most cytocompatible materials were chosen for further characterizations using Scanning electron microscopy with energy dispersive X-ray spectroscopy (SEM-EDX) and inductively coupled plasma optical emission spectroscopy (ICP-OES). Thereafter, *in vivo* investigations were carried out with the focus on material degradation, bone formation, osteointegration and the material-associated immune response during the bone healing process using established and standardized methodologies (Sieger et al., 2019; LINDNER et al., 2020a; Oberdiek et al., 2021; Pröhl et al., 2021). Finally, the correlation coefficient between the above parameters was calculated to uncover the relationship between the BBS and bone regeneration.

## 2 Materials and methods

### 2.1 Material preparation

The biphasic bone substitute (BBS) material *maxresorb* ^*®*^ (Botiss biomaterials GmbH, Berlin, Germany) composed of 60% hydroxyapatite (HA) und 40% β-tricalcium phosphate (*β*-TCP) is applied as the basis inorganic material in this study (Weiss et al., 1999). The manufacturing process of *maxresorb®* included a controlled precipitation process of aqueous solutions of calcium and phosphate as well as subsequent cold isostatic pressing into mechanically stable objects. This process results in an interconnecting porous system with defined pore diameters ranging from 200 to 800 μm, as well as micropores of 1–10 μm (Weiss et al., 1999; Barbeck et al., 2020). The further functionalization of BBS with bioactive cations and PLGA was achieved by dip-coating.

Briefly, SrCl_2_ solutions and MgCl_2_ solutions in different concentrations (0.01 M and 0.1 M) were prepared with ddH_2_O. BBS materials were incubated with each salt solution (0.2 g/mL) for 16 h at room temperature with gentle shaking, followed by drying in the drying oven at 120°C until the solution evaporated.

Sample containing PLGA coating were prepared using the following procedure: 22.5 mL of PLGA solution in acetone (2 mg/mL) were added with 1.25 mL of SrCl_2_ solutions and MgCl_2_ solutions (0.1 M), then homogenized by vortex mixing. The final concentration of Mg^2+^/Sr^2+^ in PLGA solution was 0.005 M 0.4 g BBS materials were infiltrated once/twice-/three times with 2 mL PLGA-Sr^2+^, PLGA-Mg^2+^, PLGA-Sr^2+^/Mg^2+^ solutions. Afterwards, materials were dried in the oven at 80°C until the solution evaporated.

### 2.2 Cell cultivation

Primary normal human osteoblasts (NHOst; Lonza) were cultured in OGM (OBM supplemented with OGM Single Quots; Lonza) and grown as monolayer cultures in T75 flasks (Greiner Bio-One). Trypsin/EDTA (Lonza) and TNS solution (Lonza) was used to subculture the cells twice a week. L929 fibroblasts (CLS Cell Lines Service GmbH) were cultivated in monolayer culture with DMEM media containing 10% FBS and supplemented with 1% glutamine (all: Sigma Aldrich). When reaching 80%–90% confluence, cells were trypsinized and subcultured. Both cell types were incubated at 37°C in an atmosphere containing 5% of CO_2_ and with a relative humidity of 95%. The cell number was determined with a Neubauer counting chamber (Paul Marienfeld).

#### 2.2.1 *In vitro* cytotoxicity test

*In vitro*, the cytotoxicity of sample eluates was investigated according to DIN EN ISO 10993-5. L929 and NHOst cells were used. The extracts of all samples were prepared before XTT test. Briefly, 100 mg of each sample was transferred to a reaction tube and then incubated in 1,000 µL culture medium with serum for 24 h at 37°C. Afterwards, the extracts were obtained from supernatant after vortexing and centrifuging at 2000 rpm for 3 min. Undiluted and diluted extracts in a ratio of 1:1 with fresh culture medium were added to a seeded cell lawn for sodium 3,3´-[1(phenylamino)carbonyl]-3,4-tetrazolium]-3is (4-methoxy-6-nitro) benzene sulfonic acid hydrate (XTT) assay. The fresh culture medium and culture medium with 1% Triton X100 were used as negative control and positive control, respectively. For XTT-testing the PromoKine Colorimetric Cell Viability Kit III was used. Cells were seeded at a concentration of 1 × 10^4^ cells/well in 96-well cell culture plates and then incubated overnight at 37°C and 5% CO_2_. After exchanging the medium with the extracts or control-media, cells were incubated for 24 h. Afterwards, XTT labelling reagent mixed with the electron coupling reagent was added to cells and incubated for 2 h until the orange dye became visible. Finally, the absorbance was measured photometrically at a wavelength of 450 nm. Each sample was measured in triplicate determinations.

### 2.3 Material characteristics

#### 2.3.1 SEM-EDX

The surface morphological characterization as well as the determination of element composition were performed with SEM-EDX by using a LEO Gemini 1,530 with a field-emission gun (Carl Zeiss AG, Jena, Germany) at the Helmholtz Zentrum Berlin (HZB). Samples were precoated with carbon before the morphological observation and elemental mapping. The applied voltage for imaging was set up to 5 kV. The Point-and-Shoot images were captured with a Thermo Noran X-ray detector and analyzed with Noran System Six (Version 4.1).

#### 2.3.2 Release kinetics

Release kinetics assessment was performed via Inductively Coupled Plasma—Optical Emission Spectrometry analyzer (Ultima 2, Horiba JY, France). Each sample was placed in a falcon (*n* = 4) and filled with 10 mL of distilled water, then kept in a drying oven at 37°C for 10 min to simulate body temperature. At the end of the time, sample were removed from the drying oven and the solution was analyzed using ICP-OES after filtering. The samples were reused for the next two upcoming time points, 24 and 168 h, under same conditions.

#### 2.3.3 FTIR-ATR

The infrared spectroscopic measurements were made with the Bruker Vertex 70 IR spectrometer with the Golden Gate Spectra reflection unit (wavenumber 400-40; resolution of 1 cm^−1^). The spectra were analyzed with the Bruker software OPUS Version 7.8.

#### 2.3.4 XRD

The phase composition and crystallinity of all samples were characterized by XRD using the Bruker D8 Advance. The measure-technique is based on a copper tube with SOL-X semiconductor detector. Bragg-Brentano geometry was used. All samples were measured with Cu-Kα-radiation at a wavelength of λ = 1.54178 Å (measurement parameters: stop 0,02°; 0,5 s). The diffracto-grams were matched with a database.

### 2.4 *In vivo* study

#### 2.4.1 Pre- and post-implantation procedure, surgical procedure

The *in vivo* study, which accorded to calvaria implantation model, was authorized by the Local Ethical Committee of the Faculty of Medicine, University of Niš, Niš, Serbia, and was based on the approval number 323-07-00073/2017-05/7 of the Veterinary Directorate of the Ministry of Agriculture, Forestry and Water Management of the Republic of Serbia (date of approval: 22 February 2017). A total of 42 male Wistar rats aged ten to 12 weeks were randomly assigned to two study groups (BBS_Sr^2+^ and BBS_PLGA_Sr^2+^/Mg^2+^, Table 1). The sample size was calculated via a power analysis with an additional drop-out rate of 5% (effect size 1.3, G*Power) (Faul et al., 2007). Each of the two groups contained 21 Wistar rats that were assigned to the groups by simple randomization method by the *in vivo* study director, as previously described (Al-Maawi et al., 2021). Wistar rats were sacrificed for each group at 3 time point (*n* = 7; 10, 30, and 90 days). Animals were housed in standard conditions, provided with regular mouse pellets, unrestricted access to water, and subjected to a 12-h artificial light-dark cycle. Animals were obtained from and kept in the Vivarium of the Faculty of Medicine, University of Niš, Serbia.

**TABLE 1:** Overview of the study groups, presenting the number of experimental animals per group at each timepoint.

	BBS_Sr^2+^	BBS_PLGA_Sr^2+^/Mg^2+^	Control group
10 days	7	7	14
30 days	7	7	14
90 days	7	7	14
Number per study group	21	21	--
	42 experimental animals in total		

**FIGURE 1:**
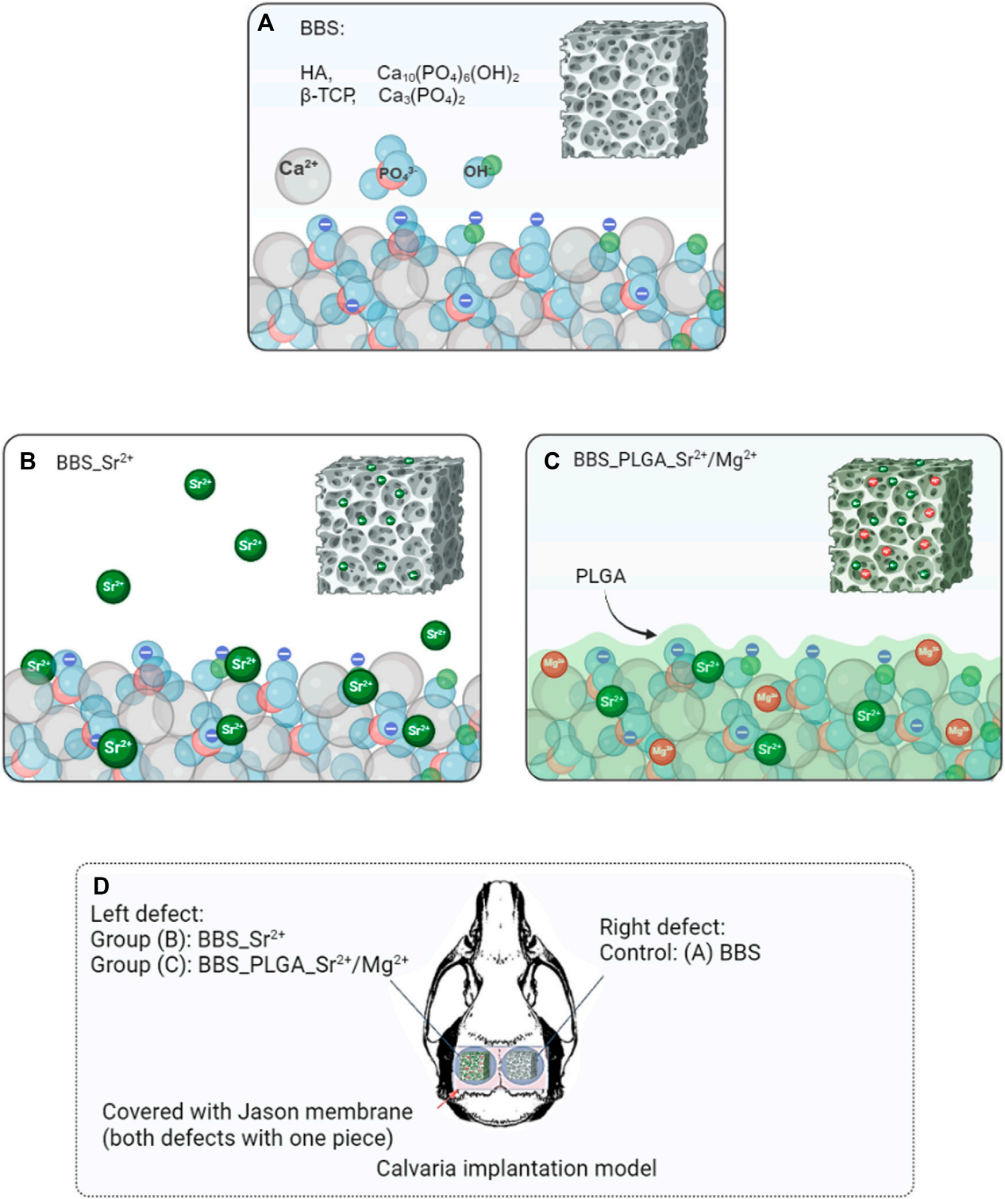
Schematic illustration of the modification processes. **(A)** The pure BBS (maxresorb^®^), composed of HA and β-TCP; **(B)** the electrostatic adsorption process; **(C)** the PLGA _Sr^2+^/Mg^2+^ coating process. **(D)** An overview of the *in vivo* study using the calvaria implantation model (bilateral defect generation) in Wistar rats.

The implantation was performed following the protocol described by Pröhl *et al.* (Figure 1) without control of confounders (Pröhl et al., 2021). In brief, the surgical area was shaved and disinfected after anesthesia. The incision was then made in the sagittal plane below the midline of the skull. Subsequently, two holes were drilled in each skull without damaging the dura mater. The coated bone substitutes were implanted in the left defect, while the commercial bone substitute Maxresorb^®^ (botiss biomaterials GmbH, Germany) was implanted in the right defect. Maxresorb^®^ was used as a control group for this study. Afterwards, both defect holes were covered by Jason membrane (botiss biomaterials GmbH, GmbH, Germany). Finally, the soft tissue was sutured via suture material (Prolene 6.0, Ethicon, Germany).

Postoperative wound openings or infections or other (inflammatory) abnormalities of the wound areas or in the behavior of the test animals, which would have been registered during the daily control of the test animals, were defined as exclusion criteria.

The explantation started after healing periods of 10, 30, and 90 days. Therefore, after the euthanasia the implanted bone substitutes were cut out with the peri-implant tissue. Each explant was finally fixed in 4% neutral-buffered formalin for 48 h.

### 2.5 Histological workup

Histological workup was performed by following the Technovit 9,100 protocol. Briefly, all explants were embedded in PMMA and sanded with sandpaper (80 grit) into a diamond shape. Tissue blocks were then cut with a thickness of 5–7 µm using a microtome (MicroTec Laborgeräte GmbH; Type: CUT4060E; S-Nr.: 601370). Movat Pentachrome-stainings, CD163-and CD11c-staining were performed following the intern protocols. Movat Pentachrome-stainings was used to quantitatively assess osteogenesis, osteoconduction and residual BBS. CD11c served as a marker for the anti-inflammatory M1 phenotype, while CD163 was utilized to identify the pro-inflammatory phenotype.

### 2.6 Histopathological analysis

The qualitative histopathological analysis was conducted by following a previously published protocol (Ghanaati et al., 2010a; Barbeck et al., 2015c; Barbeck et al., 2015d; Barbeck et al., 2016b). Parameters like cells participating in the integration and degradation process of the bone substitutes or inflammatory tissue reactions were analyzed using a light microscope (Axio Imager A2, Carl Zeiss Microscopy GmbH, Germany). Histological images were taken by using Axiocam 506 color of Carl Zeiss Microscopy GmbH, Germany.

### 2.7 Histomorphometrical analysis

Using ImageJ (Version 1.52t), all stained slides digitized with PreciPoint M8 microscope were analyzed to obtain results on bone growth, osteoconductivity and macrophage subtypes.

To evaluate bone growth and residual BBS, the ROI (Region of Interest) for total area of each defect area [in µm^2^], newly formed bone area [in µm^2^] and the bone substitute material area [in µm^2^] was manually marked in ImageJ and then measured automatically. The percentage of newly formed bone and bone substitute to the total area were evaluated for each biomaterial, defect and timepoint.

To assess osteoconductivity, the total distance of new bone - bone substitute surface—interaction [in µm] was measured for each biomaterial, timepoint and defect area. The calculated values were related to the total circumference of all bone substitute granules [in µm] located in the delimited defect area.

To determine the number of M1 and M2 macrophages, the soft tissue in the defect area was delimited manually. Then, a specialized plugin was used to count the number of stained cells within delimited total area automatically, which was described by Lindner *et al.* (Lindner et al., 2020b). Finally, the number of cells per mm^2^ was obtained. In addition, CD11c-positive MNGCs were counted manually.

### 2.8 Statistical analysis

The histomorphometrical data underwent normality testing using the Shapiro–Wilk test and variance homogeneity testing using the Brown-Forsythe test.

Subsequently, the data underwent analysis of variance (ANOVA), an extension of the T-test, for statistical analysis. A Tukey post-hoc assessment followed to compare groups by using GraphPad Prism software (Version 9.0.0, GraphPad Software Inc., La Jolla, United States). The statistical differences were defined by three significance levels. If *p*-values were less than 0.05 (#/*p < 0.05), the difference were considered as significant. High significant were considered, if the *p*-values were less than 0.01 (##/**p < 0.01) or less than 0.001 (###/***p < 0.001). By using GraphPad Prism software, the data were represented as average values and standard deviation.

Furthermore, multiple variable correlation analyses were conducted based on the measurements within the BBS group in the present study. Correlation matrix analyses using a two-tailed Pearson’s correlation were conducted to identify any possible relationships between the two parameters within the BBS group. Pearson’s correlation coefficient (r) was used to determine the strength and direction of the correlation, whether it was positive, negative, or weak. The confidence interval was set at 95%.

## 3 Results

### 3.1 *In vitro* cytocompatibility test

To evaluate the cytocompatibility of the BBS combined with 18 coatings based on the bioactive cations strontium (Sr^2+^) and magnesium (Mg^2+^) as well as PLGA coating, XTT cell viability assays were conducted using L929-fibroblasts and Normal Human Osteoblasts (NHOsts) following the DIN ISO 10993-5 protocol as previously described (Gogele et al., 2020; Schroepfer et al., 2020; Pantermehl et al., 2022; Ren et al., 2023). Based on the cytocompatibility analyses, the material types with a viability above 70% compared to the negative control group as defined within the DIN ISO norm were considered cytocompatible and used for further material characterizations and *in vivo* studies.

The results of this initial analysis step revealed that only the material combination that combined the BBS with the single PLGA coating including MgCl_2_ and SrCl_2_ showed completely adequate cytocompatibility values in case of both cell lines above 70% and in both extract conditions (Table 2). Additionally, the material combination of the BBS coated with a tenfold dilution of SrCl_2_ could be considered cytocompatible as it induced only low cytotoxicity for NHOsts in contact with the 100% extracts, while the other values were in the non-cytotoxic range (Table 2). All other material combinations showed more or higher deviations from the non-cytotoxicity ranges for both both cell types or the different extract concentrations and were therefore classified as cytoincompatible (Table 2). Based on these results both material combinations, i.e., the BBS coated with a tenfold dilution of SrCl_2_ (BBS_Sr^2+^) and the BBS with the single PLGA coating including MgCl_2_ and SrCl_2_ (BBS_PLGA_Sr^2+^/Mg^2+^), were chosen for the next study step.

**TABLE 2:** An overview of the results of the cytotoxicity testing using L929 fibroblasts and NHOsts.

BBS with 30 coatings	Cytotoxicity (compared with negative control in %)			
	L929 fibroblasts		NHOsts	
	100% of extracts	50% of extracts	100% of extracts	50% of extracts
1. BBS_MgCl_2_	55.7	76.8	30.7	52.2
2. BBS_SrCl_2_	60.8	75.8	95.7	109.0
3. BBS_MgCl_2_-SrCl_2_	56.5	72.0	−6.4	95.7
4. BBS_MgCl_2_ (0.1x)	67.5	91.0	63.1	82.4
5. BBS_SrCl_2_ (0.1x)	80.8	96.0	61.0	78.7
6. BBS_MgCl_2_-SrCl_2_ (0.1x)	75.6	73.5	51.7	63.7
7. BBS_MgHPO_4_	72.3	67.9	31.7	39.0
8. BBS_ SrHPO_4_	71.7	66.4	53.3	66.7
9. BBS_MgHPO_4_-SrHPO_4_	61.9	62.0	36.4	41.7
10. BBS_PLGA_MgCl_2__1	73.2	65.5	39.5	53.5
11. BBS_PLGA_MgCl_2__2	31.2	74.6	26.4	57.3
12. BBS_PLGA_MgCl_2__3	23.9	73.0	19.2	40.0
13. BBS_PLGA_SrCl_2__1	111.7	104.6	52.3	72.7
14. BBS_PLGA_SrCl_2__2	51.0	58.3	102.9	106.7
15. BBS_PLGA_SrCl_2__3	5.7	79.2	4.9	56.9
16. BBS_PLGA_MgCl2-SrCl_2__1	77.1	89.5	72.2	88.8
17. BBS_PLGA_MgCl2-SrCl_2__2	39.2	62.5	29.6	54.7
18. BBS_PLGA_MgCl2-SrCl_2__3	−0.8	73.0	2.2	43.9

PLGA, poly (lactic-co-glycolic acid); 1/2/3 = Single/dual/triple coated; 0.1x = Tenfold dilution of 0.1M salt solution; Green coloring = cell viability above 70%. Green groups = selected group.

### 3.2 Material characteristics

#### 3.2.1 Scanning electron microscopy with energy dispersive X-ray spectroscopy (SEM-EDX)

**FIGURE 2:**
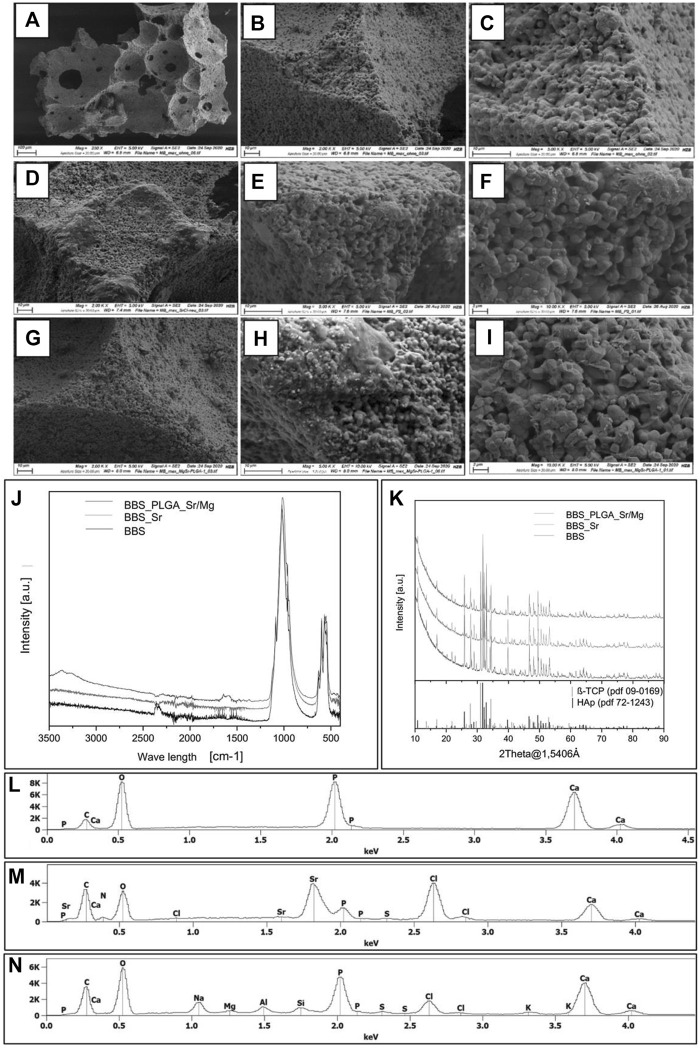
**(A–I)** SEM images of bare BBS **(A,B,C)**, BBS coated with the Sr^2+^ solution **(D,E,F)**, and the BBS combined with the coating based on PLGA, MgCl_2_ and SrCl_2_ **(G,H,I)**. **(J)** FTIR spectra of all bone substitutes, **(K)** XRD diffractograms of all bone substitutes, **(L)** EDX spectrum of the pure BBS, **(M)** EDX spectrum of the BBS_Sr^2+^ group and **(N)** EDX spectrum of the BBS_PLGA_ Sr^2+^/Mg^2+^ group.

SEM images of pure BBS and both coated BBS compositions, i.e., the BBS coated with a tenfold dilution of SrCl_2_ and the BBS with the single PLGA coating including MgCl_2_ and SrCl_2_, were depicted in Figure 2. The BBS has an open pore structure (Figures 2A–C) (Konermann et al., 2014). After the coating with the Sr^2+^ ion solution, the material showed a more even “fused” surface structure compared to the bare BBS, which indicates an effective coating adhesion (Figures 2D–F). In addition, highly grown and superimposed elongated crystallites were observed at the material surfaces. The SEM analysis of BBS with the single PLGA coating including MgCl_2_ and SrCl_2_ revealed that this material composition had a smooth and continuous surface, which could be associated with the PLGA polymer layer (Figures 2G–I). Thereby, the micro-structure of the BBS granules was no longer present in form of regular spherical particles as in case of the pure BBS but showed a cubic or polyhedral structure (Figure 2I). Overall, both coating techniques were effectively processed and could be verified via SEM on the surface of the BBS granules. Moreover, none of the coatings did change the porosity of the BBS.

The results of SEM-EDX elemental analysis are shown in Figures 2L–N and Table 3. Calcium (Ca), phosphate (P) and oxygen (O) were observed in all analyzed biomaterials indicating the existence of the calcium phosphate basis of the BBS. The weight percentage of Ca, P and O in the group of the bare BBS was higher than in case of the materials coated with both different methodologies. Furthermore, the BBS coated with the Sr^2+^ ion solution exhibited a high weight percentage of strontium (Sr, 27.73 ± 0.21 wt%) and chloride (Cl, 24.06 ± 0.20 wt%), suggesting the successful addition of Sr^2+^ on the surfaces of the BBS. The BBS combined with the single PLGA coating including MgCl_2_ and SrCl_2_ presented a higher composition of Ca, P, O than the BBS coated with the Sr^2+^ solution. Moreover, the weight percentage of Sr^2+^ present in the PLGA coated BBS was comparable lower.

**TABLE 3:** EDX analysis of bare BBS, BBS_Sr^2+^ and BBS_PLGA_Sr^2+^/Mg^2+^.

	O-K	P-K	Ca-K	Sr-L	Mg-K	Cl-K
BBS	39.1 ± 0.24	18.2 ± 0.14	42.8 ± 0.26	—	—	—
BBS_Sr^2+^	23.2 ± 0.23	4.8 ± 0.14	20.1 ± 0.16	27.7 ± 0.21	—	24.1 ± 0.20
BBS_PLGA_Sr^2+^/Mg^2+^	40,0 ± 0.33	13.6 ± 0.12	29.2 ± 0.29	—	0.5 ± 0.04	10.8 ± 0.14

#### 3.2.2 Release kinetics

**FIGURE 3:**
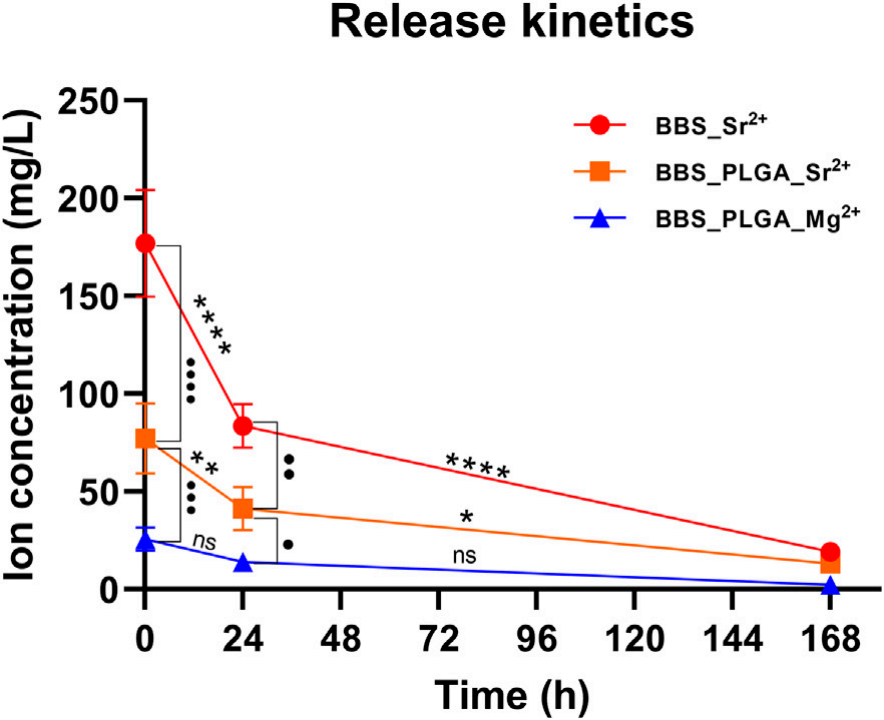
Ion release profile of BBS_Sr^2+^ group, and BBS_PLGA_Mg^2+^/Sr^2+^ group determined by ICP-OES (Mean ± SD, *n* = 4, intra-/inter-individual significances: ns = no significance, */• *p* < 0.05, **/•• *p* < 0.01, ••• *p* < 0.001, ****/•••• *p* < 0.0001).

Both coated BBS materials displayed similar trends in the release of Sr^2+^ ions (Figure 3). Thus, the initial release of Sr^2+^ ions was significantly high within the first 24 h (**p < 0.01, ****p < 0.0001) in both study groups, followed by a gradual significant decrease in both study groups until 168 h (*p < 0.05, ****p < 0.0001) (Figure 3). In the group of the BBS with the PLGA coating including MgCl_2_ and SrCl_2_, the concentrations of both Mg^2+^ and Sr^2+^ ions in preparation solution were 0.005 M, while the Sr^2+^ concentration in the BBS_Sr^2+^ group was 0.01 M. As a result, the group with BBS coated with the Sr^2+^ solution exhibited the greatest Sr^2+^ ion release at all three time points, significantly surpassing the levels observed in the BBS_PLGA_ Sr^2+^/Mg^2+^ group (•• *p* < 0.01, •••• *p* < 0.0001).

Although the release of Mg^2+^ ions also showed a decreasing trend over time, no significant differences were observed at the three time points. Moreover, the release of Sr^2+^ ions in the BBS_PLGA_Sr^2+^/Mg^2+^ group was about 3 times significantly higher than that of Mg^2+^ ions (•• *p* < 0.01, ••• *p* < 0.001). At 168 h, the metal ions in both groups approached complete release, with no observed intergroup significances.

### 3.3 *In vivo* studies

For the *in vivo* study, the coated bone substitutes were implanted into the left defect in Wistar rats, while the commercial bone substitute Maxresorb^®^ (botiss biomaterials GmbH, Germany) was implanted into the right defect.

#### 3.3.1 Histopathological analysis

**FIGURE 4:**
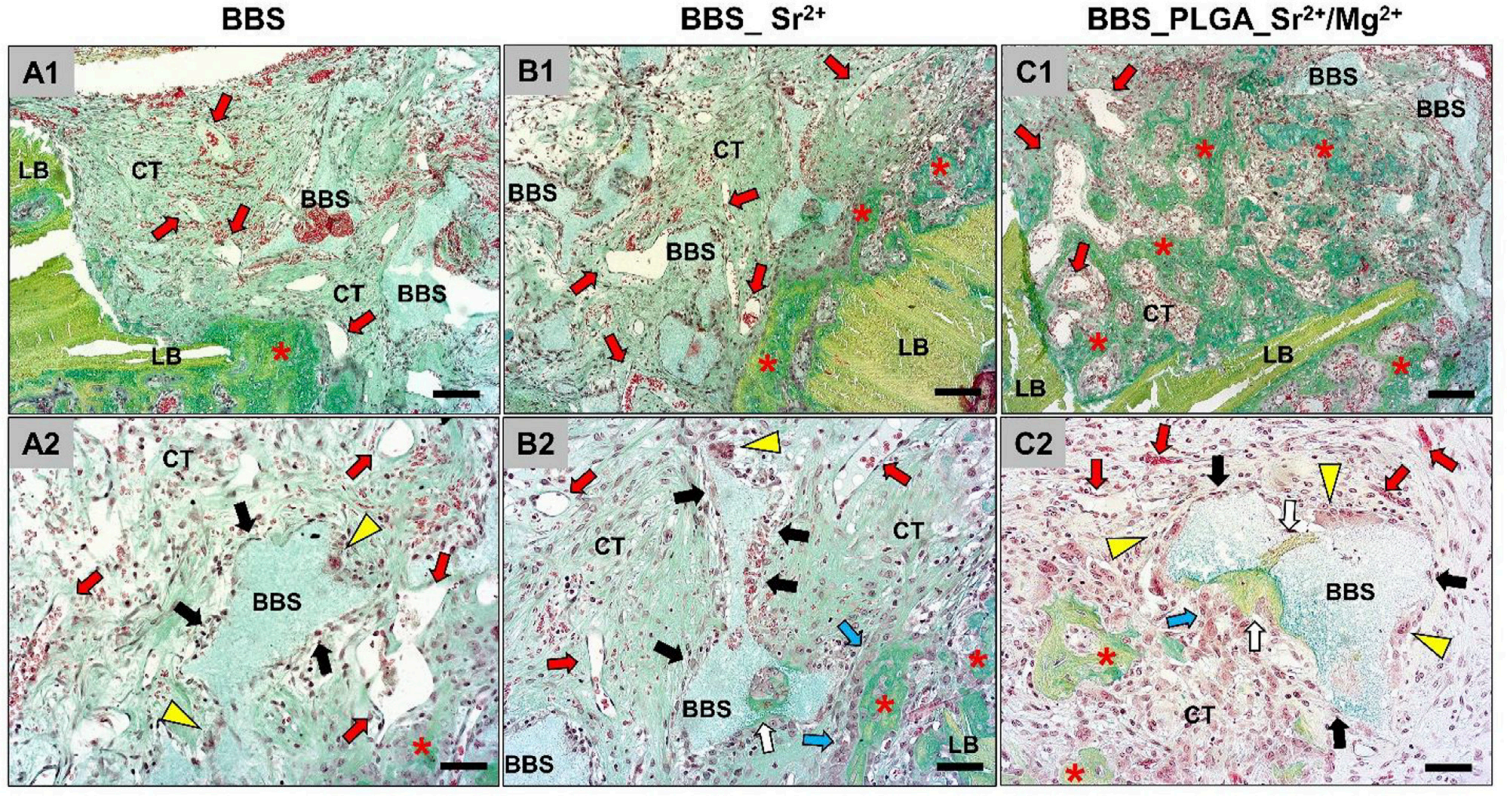
An overview of the implantation areas in all study groups at day 10 post-implantation and the tissue reactions to the BBS granules. BBS = granules of the biphasic bone substitute, LB = local bone, CT = connective tissue, red stars = newly formed bone matrix, red arrows = blood vessels, white arrows = new material-related bone, black arrows = macrophages, yellow arrowheads = multinucleated giant cells, blue arrows = osteoblasts (Movat Pentachrome-stainings, **(A1–C1)**, ×100 magnification, scale bar = 50 μm; **(A2–C2)**, ×200 magnification, scale bar = 20 µm).

At day 10 post-implantation (Figure 4), the implanted bone substitute granules in all study groups could be localized within the defect areas and were mostly embedded within connective tissue.

Inflammatory cells, i.e., mainly macrophages as well as low numbers of multinucleated giant cells, could be identified at the surfaces of the BBS granules without visible differences between the study groups. Moderate numbers of lymphocytes and granulocytes were additionally found within the intergranular connective tissue that showed a moderate vascularization in comparable extents in all study groups. In addition, slight bone growth was visible at the defect margins in form of uncalcified bone matrix, which was associated with osteoblastic hems. Thereby, a material-dependent bone growth was less detectable than a material-independent matrix growth outgoing from the local residual bone at this early study time point. In summary, no differences in the extents of the material-related tissue reactions could be registered.

**FIGURE 5:**
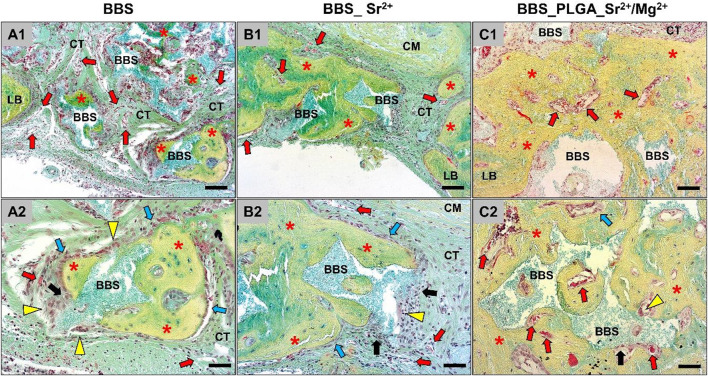
An overview of the implantation areas in all study groups at day 30 post implantation and the tissue reactions to the bone substitute granules. BBS = granules of the biphasic bone substitute, LB = local bone, CT = connective tissue, red stars = newly formed bone matrix, red arrows = blood vessels, black arrows = macrophages, yellow arrowheads = multinucleated giant cells, blue arrows = osteoblasts (Movat Pentachrome-stainings, **(A1–C1)**, ×100 magnification, scale bar = 50 μm; **(A2–C2)**, ×200 magnification, scale bar = 20 µm).

At day 30 post-implantation, the histological evaluation showed that more newly formed bone tissue could be detected particularly within the peripheral defect areas close to the defect borders (Figure 5).

In all three study groups, the material particles (BBS) were embedded in newly formed bone matrix within the defect areas showing a material-dependent bone growth. Still, less bone growth within the central defect area in all study groups was detectable. At this post-implantation time point parts of the newly formed bone matrix were not fully calcified and remained associated with many osteoblastic hems indicating further bone tissue development. Also, a cell- and vessel-rich connective tissue was detected within the granular interspaces, which was mainly composed of macrophages between lower numbers of lymphocytes, and fibroblasts. At this time point, mainly macrophages as well as lower numbers of multinucleated giant cells could be detected at the material surfaces in all study groups in comparable extents. In summary, no differences in the extents of the material-related tissue reactions between the study groups could be observed.

**FIGURE 6:**
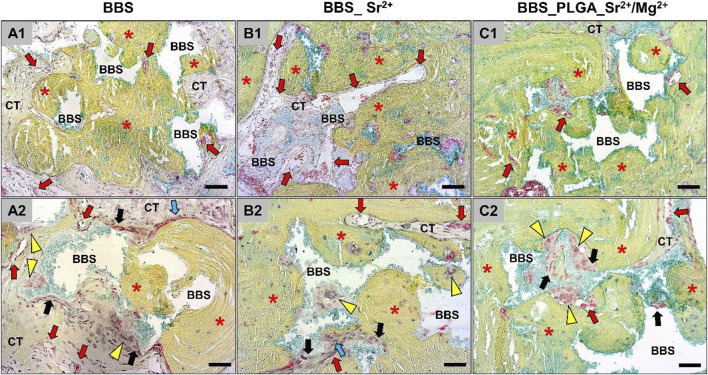
An overview of the implantation areas in all groups at day 90 post implantation and the tissue reaction to the bone substitutes granules. BBS = granules of the biphasic bone substitute, LB = local bone, CT = connective tissue, red stars = newly formed bone matrix, red arrows = blood vessels, black arrows = macrophages, yellow arrowheads = multinucleated giant cells, blue arrows = osteoblasts (Movat Pentachrome-stainings, **(A1–C1)**, ×100 magnification, scale bar = 50 μm; **(A2–C2)**, ×200 magnification, scale bar = 20 µm).

At day 90 post-implantation, a higher extent of newly formed bone matrix was detectable in all three study groups up to the central defect areas (Figure 6).

In most of the defect areas a complete bony regeneration was detectable, and all granules of the biphasic bone substitutes were embedded within newly bone matrix at this late study time point. Furthermore, the histological analysis indicated that even at this late study time point, osteoblastic hems were detectable, which indicated a continued bone formation. Also, a cell- and vessel-rich connective tissue was detected, which was still mainly composed of macrophages and fibroblasts. High numbers of macrophages between lower numbers of multinucleated giant cells were still detected on the surfaces of the material granules that were neighbored to connective tissue (Figure 6). In summary, no differences in the extents of the material-related tissue reactions between the three study groups can be registered.

**FIGURE 7:**
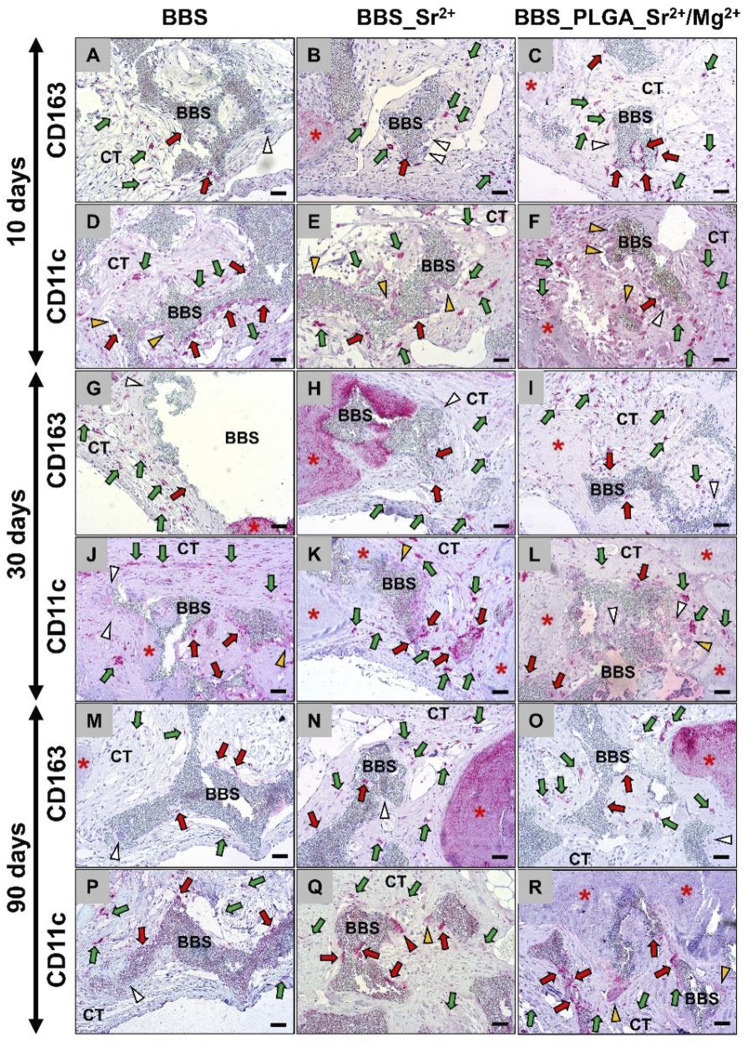
Exemplary histological images of the immune response via immunodetection of anti-(CD163)- and pro-(CD11c)-inflammatory macrophages in the implantation areas of all groups at the **(A–F)** 10, **(G–L)** 30 and **(M–R)** 90 days post-implantation. BBS = bone substitute material, red stars = newly formed bone matrix, green arrows = positive cells within the intergranular connective tissue, red arrows = positive cells at the material surfaces, white arrowheads = negative multinucleated giant cells, orange arrowheads = weakly positive multinucleated giant cells, red arrowheads = positive multinuclear giant cells **(A–C,G–I,M–O)**: immunohistochemical CD163 detection and **(D–F,J–L,P–R)**: immunohistochemical CD11c-detection; ×200 magnification, scale bar = 20 µm).

The histopathological results of the macrophage subpopulations revealed that considerable numbers of pro-inflammatory M1-and anti-inflammatory M2-macrophages were detectable at all implantation sites (Figure 7).

Thereby, the numbers of pro-inflammatory macrophages were tending to be higher than the numbers of the anti-inflammatory subtype at all study time points and in all groups. Only at day 30 post-implantation the occurrence of M1-macrophage was visibly higher in all study groups compared to the other study time points. Moreover, no visible differences in the induction of both macrophage subtypes in the three different study groups were seen at any observation time point. Additionally, the observation showed that none of the material-associated multinucleated giant cells showed signs of a CD163-expression in any of the study groups, while they showed low-grade signs of an CD11c-expression. However, most of these cells were not expressing CD163 nor CD11c.

#### 3.3.2 Histomorphometrical analysis

##### 3.3.2.1 Material degradation

**FIGURE 8:**
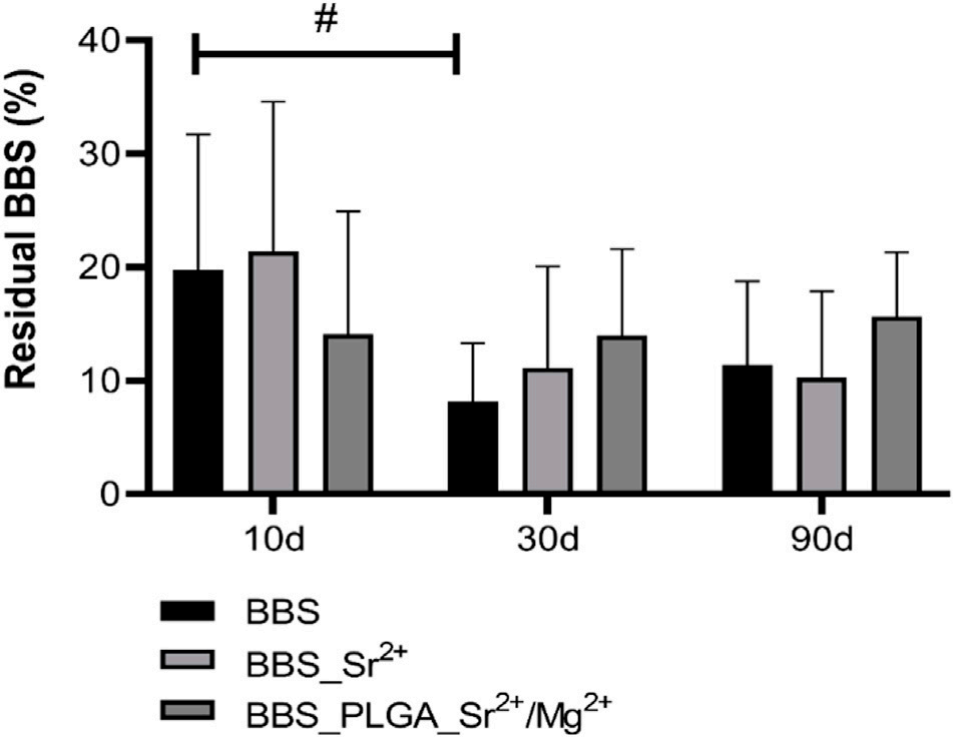
Results of the histomorphometrical analysis of the material degradation (intraindividual significances: #*p* < 0.05). The residual BBS (%) is derived by quantifying the area of BBS as a proportion of the defect area.

The histomorphological analysis of material degradation assessed the granule area as a percentage of the total implanted region. The findings indicated a reduction of the granule sizes in both the BBS and BBS_Sr^2+^ group over the study period, whereas the granule size in the BBS_PLGA_Sr^2+^/Mg^2+^ group remained consistent throughout the implantation period (Figure 8).

In particular, the granule size in the BBS group was significantly decreasing between day 10 and 30 days (#*p* < 0.05, Figure 8), while only a trend towards a material degradation within this time frame was found in the BBS_Sr^2+^ group. Between day 30 and 90 post-implantation no further material degradation was measured. Moreover, no significant or tending differences in the granule sizes were found in the BBS_PLGA_Sr^2+^/Mg^2+^ group at all three study time points.

##### 3.3.2.2 Bone regeneration and osteoconductivity

**FIGURE 9:**
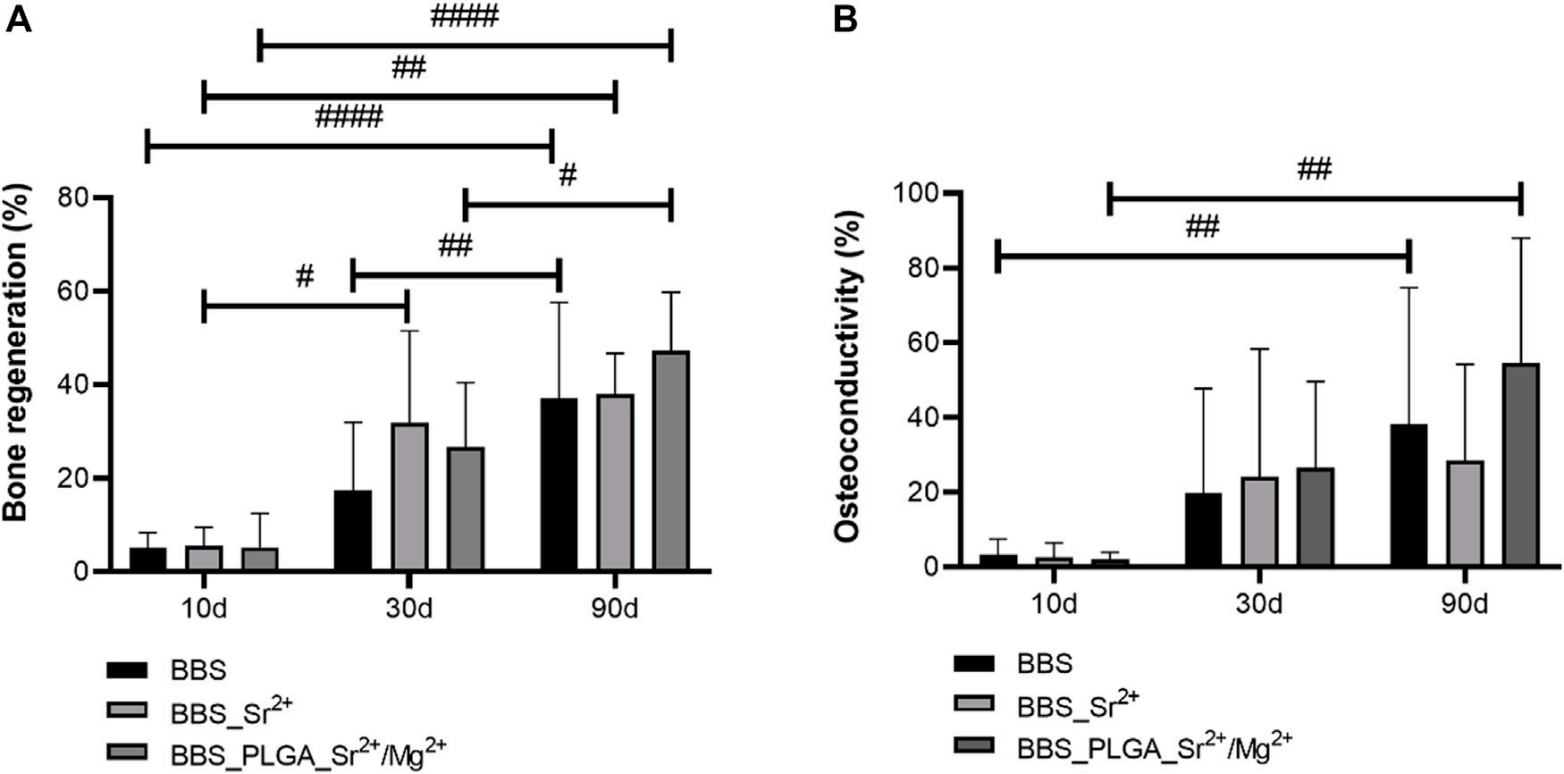
Results of the histomorphometrical analyses of **(A)** bone regeneration and **(B)** osteoconductivity (intraindividual significances: #*p* < 0.05, ##*p* < 0.01, ####*p* < 0.0001).

The histomorphometrical analysis of the bone regeneration, measuring the new bone area as a percentage of the total implanted region, indicated that no interindividual differences between the amounts of newly formed bone between the three study groups were found at any of the observation time points (Figure 9A).

Comparable regeneration patterns were observed in all groups starting with small amounts of newly formed bone of approximately 5% in all groups at day 10 post-implantation. At day 30 post-implantation also comparable bone regeneration pattern were found in all groups (Figure 9A). The highest amount of bone formation was found in the BBS_Sr^2+^ group (31,78% ± 17.11%), while lower bone formation amount was measured in the BBS_PLGA_Sr^2+^/Mg^2+^ group (26.76% ± 12.25%) and the lowest amount was detected in the control BBS group (17.46% ± 13.08%) but without significant differences (Figure 9A). At day 90 post-implantation still comparable bone regeneration pattern were found in all groups with the highest bone formation in the BBS_PLGA_Sr^2+^/Mg^2+^ group (47.36% ± 11.36%) and approximately equal lower values in the BBS_Sr^2+^ group (38.04% ± 7.54%) and the control BBS group (37.05% ± 19.52%) but still without significant differences (Figure 9A).

At day 30 post-implantation only in the BBS_Sr^2+^ group a significant increase of bone formation was found compared to day 10 (#*p* < 0.05, Figure 9A). At day 90 post-implantation significant increases of bone regeneration compared to day 30 were detected in the control BBS group and the BBS_PLGA_Sr^2+^/Mg^2+^ group (#*p* < 0.05 and ##*p* < 0.01, Figure 9A). Moreover, all experimental groups exhibited significantly higher bone regeneration at day 90 post-implantation in comparison to the values at day 10 (##*p* < 0.01 and ####*p* < 0.0001, Figure 9A).

The histomorphometrical analysis of the osteoconductivity revealed that no significant differences among the three groups at any of the study time points (Figure 9B). At day 90 post-implantation in the BBS group and the BBS_PLGA_Sr^2+^/Mg^2+^ group significantly higher osteoconductivity in comparison to the values at day 10 were measured (##*p* < 0.01) (Figure 9B).

##### 3.3.2.3 Immune response

**FIGURE 10:**
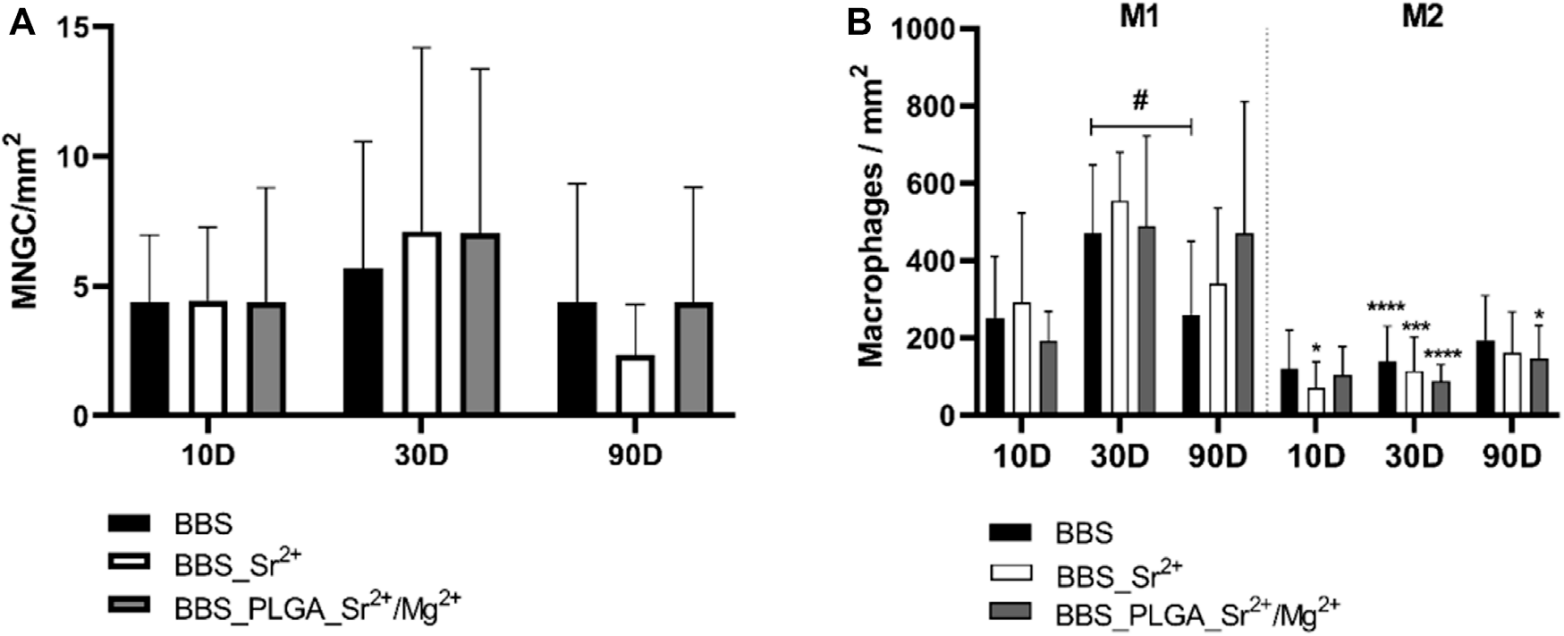
Results of the histomorphometrical analyses of the occurrence of **(A)** CD11c-positive multinucleated giant cells and **(B)** pro- and anti-inflammatory (M1 and M2) macrophage subtypes (intraindividual differences: #/*p < 0.05, ***p < 0.001, ****p < 0.0001. * Indicates significance between M1 and M2).

The histomorphometrical evaluation of the occurrence of CD11c-positive multinucleated giant cells showed the absence of significant differences between the three study groups at any of the observation time points (Figure 10A).

A tendency towards an increase of the occurrence of CD11c-positive multinucleated giant cells was observed in all study groups at day 30 post-implantation, while a further trend towards a decrease up to day 90 post-implantation was also detectable—especially in the BBS_Sr^2+^ group (Figure 10A).

The histomorphometrical assessment of the macrophage subtypes revealed similar macrophage responses in all study groups and at all time points characterized by a prevalence of pro-inflammatory macrophages over the anti-inflammatory counterpart (Figure 10B). At day 10 post-implantation only in a significantly higher number of pro-inflammatory macrophages compared to the anti-inflammatory subtype in the BBS_Sr^2+^ group (*p < 0.05) was measured. At day 30 post-implantation significantly higher numbers of pro-inflammatory macrophages compared to the anti-inflammatory subtypes were found in all study groups (***p < 0.001, ****p < 0.0001) (Figure 10B). Finally, significantly higher values of pro-inflammatory macrophages (*p < 0.05) were also found in the BBS_PLGA_Sr^2+^/Mg^2+^ group at 90 days post-implantation. Only between day 30 and 90 post-implantation a significant decrease of the number of pro-inflammatory macrophages in the BBS group was found (##*p* < 0.01) (Figure 10B).

##### 3.3.2.4 Correlation analysis

**FIGURE 11:**
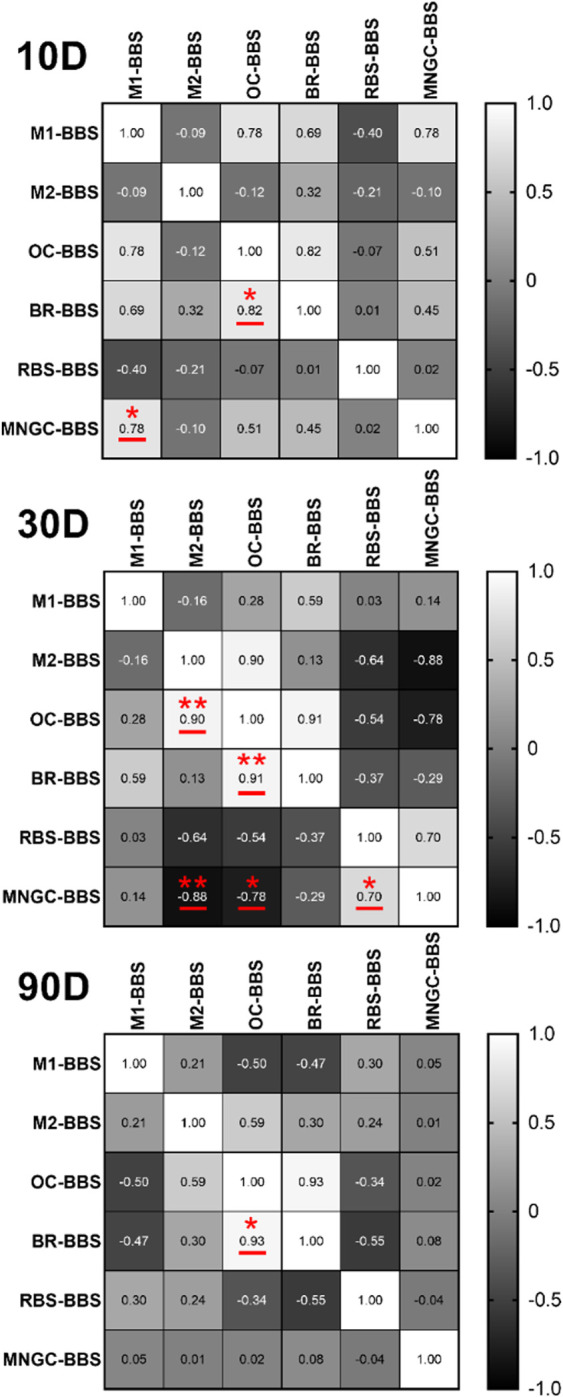
Correlation matrices between all measured variables of BBS group. The values in the matrix are the correlation coefficients r: weak correlation (0 ≤ |r| < 0.4); moderate correlation (0.4 ≤ |r| < 0.7); strong correlation (0.7 ≤ |r| < 1) (intraindividual significanes: *p < 0.05, **p < 0.01). M1 = pro-inflammatory macrophages, M2 = anti-inflammatory macrophages, OC = osteoconductivity, BR = bone regeneration, RBS = residual bone substitute, MNGC = multinucleated giant cells.

Pearson’s correlation analysis was utilized to explore the relationships between the five measured parameters. Only in the BBS groups significant correlations were found (Figure 11).

At all timepoints, a significant positive correlation (*p < 0.05, **p < 0.01) was observed between osteoconductivity (OC) and bone regeneration (BR) (Figure 11). Although not statistically significant, there was tendentially a moderate positive correlation between M1 and BR at day 10 and 30 post-implantation, but a negative correlation at 90 days.

Additionally, at day 10 post-implantation, a positive correlation between the occurrences of MNGCs and M1-macrophages was observed (*p < 0.05). At day 30 post-implantation, a positive correlation (**p < 0.01) between the occurrence of M2-macrophages and osseoconductivity was calculated. Addionally, different correlations between the occurrence of MNGCs and different other paramters were found (Figure 11). A positive significant correlation of the MNGC occurrence und the amount of the remaining BBS (*p < 0.05) was measured. Moreover, negative correlations between the MNGC occurrence and the occurrence of M2-macrophages (**p < 0.01) but also the osseoconductivity (*p < 0.05) were measured.

## 4 Discussion

Even after several decades of research in the field of bone substitutes and a broad variety of material developments and biological studies focusing on new synthetic materials as an alternative to autologous bone transplants, this topic is still current. It is important to find material factors that combine both the osseoregenerative properties that optimally support tissue regeneration and, on the other hand, the material-induced immune responses that optimally support healing cascades but also lead to a material durability in the sense of *restitution ad integrum*.

The cytocompatibility of newly developed biomaterials is an important step to consider the further development and analysis of biomaterials for biomedical applications. The results of the XTT cell viability assay indicated that most of the coatings including the bioactive cations on BBS exhibited cytotoxicity, while only a few of the material prototypes were cytocompatible. Among the four materials selected based on suitable cytocompatibility values using L929 cells, only one material, BBS_PLGA_MgCl_2_-SrCl_2_, showed an adequate cell viability of NHOst cells above 70%. This result suggests that this material prototype may have the potential to promote cell growth and proliferation, making it a promising candidate for use in tissue engineering applications. Additionally, the material prototype BBS_SrCl_2_ (10x) showed only slight cytotoxicity on NHOst cells, thus it was also selected for the further physiochemical experiments and the *in vivo* study part. Overall, these results highlight the importance of a safe and effecting loading strategy.

The results of the SEM analysis and the EDX elemental analysis indicate that the coating techniques were effectively processed as the coatings were detectable at the surface of the BBS but did not change the material structure. The higher Ca, P and O compositions in the BBS_PLGA_Sr^2+^/Mg^2+^ group should be regarded as a result of the PLGA coating. Furthermore, the exceptionally low strontium content in this group may be a consequence of either non-uniform distribution or concealment by the PLGA coating. The PLGA coating was originally introduced to improve the bio-performance and long-term ion release kinetic of BBS. Nonetheless, the present findings indicate that the Sr^2+^ ion release patterns in case of the BBS_PLGA_Sr^2+^/Mg^2+^ and the BBS_Sr^2+^ prepared are comparable. The BBS_PLGA_Sr^2+^/Mg^2+^ group exhibited a lower release of strontium ions at all timepoints compared to that in the BBS_Sr^2+^ group, which can primarily be attributed to the distinct initial loadings. Notably, magnesium ions are released more evenly in the BBS_PLGA_Sr^2+^/Mg^2+^ group, which was significantly lower than the release of strontium ions at all time points. This observation may be explained on the one hand by the smaller ionic radius and higher charge density of magnesium ions, which might facilitate their enhanced adsorption onto the BBS surface and stronger binding to the carboxyl group of PLGA. On the other hand, it is also plausible that the initial loading of magnesium ions onto the BBS is lower, consequently leading to reduced release.

The histopathological findings from the *in vivo* study part revealed a comparable moderate foreign body reaction in response to implantation of all bone substitute granules. The tissue response was mainly characterized by the presence of macrophages and lower numbers of granulocytes, material-associated multinucleated giant cells (MNGCs), and lymphocytes, which is a common response to materials of this class but also to different other biomaterials mostly being a correlate of the cellular biodegradation (Ghanaati et al., 2010b; Barbeck et al., 2015a; Barbeck et al., 2015b; Barbeck et al., 2016a; Abels et al., 2021). Moreover, the presence of both macrophages and MNGCs can contribute to tissue healing processes due to their pro- and anti-inflammatory nature (Miron and Bosshardt, 2018). It is worth to mention that the formation of a well vascularized granulation tissue starting with day 10 up to day 90 post-implantation is not only a histopathological sign of the ongoing material degradation but also an optimal basis for tissue regeneration. This is attributed to molecules such as the expression of the vascular endothelial growth factor (VEGF) by the associated phagocytes and the related implantation bed vascularization (Ghanaati et al., 2010a; Barbeck et al., 2021). In this context it has been described that the β-TCP fraction of the BBS induces a rapid vascularization of the implantation bed based on a fast cellular degradation beside its high solubility (Ghanaati et al., 2012). At day 10 and 90 post-implantation mostly tendencies of M1 macrophages higher occurrence with few exceptions were found, while at day 30 post-implantation significantly higher numbers of M1-macrophages were observed. These might reflect the observations of the afore-mentioned publication by Ghanaati *et al.* that described the “separated” degradation pattern of both calcium phosphate compounds, i.e., the faster biodegradation pattern of the β-TCP component and the delayed reactivity of HA (Ghanaati et al., 2010a; Ghanaati et al., 2012). The histomorphometrical analysis of the residual BBS revealed that this degradation pattern was also detectable in case of the pure BBS showing a significant decrease between day 10 and 30 post-implantation, while its degradation reached a steady state up to day 90 post-implantation. Interestingly, only a tendency towards a comparable degradation pattern was found in both groups of the coated materials, but without significances. This result could be seen as an indication of an influence of the coating on the degradation behavior of the materials - initially independent of the degradation pathway, i.e., the solubility behavior and the cellular-based degradation via phagocytosis.

Based on this initial observation an analysis of and the occurrence of MNGCs was also conducted. The histomorphometrical analysis of the MNGC occurrences revealed no intra- or interindividual significances but showed a tendency towards higher numbers at day 30 post-implantation, which is comparable to the results found in case of the M1-macrophages and might underline the afore-mentioned theory of the predominant cellular biodegradation pattern of the BBS. Moreover, the correlation analysis revealed another interesting result as the occurrence of M1-macrophages and that of (pro-inflammatory) MNGCs were positively associated. This observation in conjunction with the fact that the material associated MNGCs expressed only the pro-inflammatory factor, confirms the earlier suggestion that the “pro-inflammatory limb” of the tissue response is responsible for material degradation (Abels et al., 2021; Barbeck et al., 2022). In addition, the correlation of these two phagocyte subtypes shows that the formation of MNGCs only occurs as part of the pro-inflammatory tissue response and can therefore also be assigned to the “foreign body giant cell type” based on these observations as described before by Barbeck *et al.* (Barbeck et al., 2017). On the other hand, this result can of course be attributed to the fact that the macrophages are the mononuclear precursor cells of the MNGCs, which then fuse in the further tissue reaction (Miron and Bosshardt, 2018). Additionally, a positive correlation between the remaining BBS and the occurrence of MNGCs was detected, which underlines the assumption that material phagocytosis is executed by pro-inflammatory MNGCs that seem to have a higher phagocytosis capacity compared to macrophages. In summary, our study reaffirms that primarily proinflammatory phagocytes, i.e., macrophages and MNGCs, are involved in material degradation, while the anti-inflammatory macrophage subtype seem to be involved in tissue regeneration and regulating cascades being located within the surrounding tissue (Abels et al., 2021; Barbeck et al., 2022; Unger et al., 2022).

The analysis of the material-mediated bone tissue healing showed that sufficient and comparable bone regeneration was achieved in all test groups. As expected, progressive bone formation was observed in all study groups up to day 90 post-implantation that was at this late time point significantly higher compared to day 10 post-implantation. It is worth to note that osseoregeneration in the BBS_Sr^2+^ group primarily occurred during the early implantation period reaching a “steady-state” at 30 days post-implantation, whereas the BBS group and BBS_PLGA_Sr^2+^/Mg^2+^ group experienced a more pronounced regeneration in the late study period. Although in the latter group a significantly higher M1-macrophage presence was detected, no correlation was found between these both parameters, so that this result can only be interpreted as a chance finding—especially in view of the fact that the anti-inflammatory macrophage phenotype might be associated with the process of (bone) tissue healing (Pajarinen et al., 2019). This assumption is also supported by the fact that similar bone tissue regeneration patterns were determined based on the measurement of osseoconductivity, but no significant differences were calculated. Moreover, the results of the correlation analysis substantiated this assumption in two different points: Initially, a positive correlation between the occurrence of M2-macrophages and osseoconductivity was found at day 30 post-implantation, which showed the linkage between the anti-inflammatory limb of the tissue reaction and (bone) tissue repair. Secondly, the analysis revealed at the same time point a significantly negative correlation between osseoconductivity and the occurrence of MNGCs. Moreover, the correlation analysis uncovered further positive correlation between the processes of new tissue formation and the osseoconductivity of the BBS. This is not surprising, as both processes should generally correlate in the case of critical-size defects and an osseoconductive bone substitute. These results merely confirm the material-based bone regeneration of the control group and thus the effectiveness of the BBS, which has already been tested many times both preclinically and clinically (Ghanaati et al., 2012; Bielenstein et al., 2022).

Notably, neither the BBS_Sr^2+^ group nor the BBS_PLGA_Sr^2+^/Mg^2+^ group affect the final bone regeneration and macrophages response compared to the BBS group. This result is inconsistent with the initial hypothesis but is in line with some previous *in vivo* experiments with metal ion doping. Barbeck *et al.* doped strontium and copper ions in bioactive glass, which also showed no effect on immune response or vascular integration patterns (Barbeck et al., 2022). It might be due to the fact that pro-inflammatory MNGCs were closely linked to material degradation, but these foreign body giant cells were not subject to regulation by Sr^2+^/Mg^2+^ ions in a manner similar to osteoclasts. Additionally, acidic degradation products of PLGA coatings have been reported to impact the deposition of calcium phosphate on the material surface (Maadani et al., 2023). Moreover, prior investigations have shown a strongly dose-dependent influence of both metal ions on bone regeneration. For example, a study conducted by Shen and others has demonstrated that high proportion of Sr doping promote M2-macrophage polarization and fast bone regeneration (Shen et al., 2022). Conversely, a concentration of 0.1 mmol/L of strontium displayed a mild effect on proliferation and osteogenic differentiation of hMSC (Schumacher et al., 2013). Moreover, it has been described that a Mg^2+^ concentration of 100 mg/L and above can promote osteogenic differentiation of MSC but also promotes the secretion of bone morphogenetic protein 2 (BMP2) by macrophages, and should induce phenotypic switching into M2-macrophages (Nie et al., 2022). Nevertheless, the most suitable ion concentrations highlighted across diverse studies exhibited considerable variation due to distinct preparation protocols, sample attributes, and experimental frameworks, thus impeding direct comparisons. While the ions loaded in this study didn't yield a substantial enhancement in biological effects on immune response or bone regeneration, it was noted that all bone materials induced balanced immune response and exhibited excellent tissue integration without any signs of fibrous encapsulation or other unfavorable tissue responses.

## 5 Conclusion

In conclusion, this study investigated the effects of loading of Sr^2+^ and/or Mg^2+^ ions onto BBS materials on cyto- and biocompatibility including the immune response and osteoregeneration. The results showed that two cytocompatible ion-modified material variants were identified and further tested by the *in vitro* cytocompatibility analysis.

All examined material groups provided a good *in vivo* biocompatibility with a moderate and balanced inflammatory response and significant new bone regeneration during the healing period. Notably, the BBS_Sr^2+^ group exhibited a distinct faster regeneration compared to the other two groups. The formation of new bone tissue predominantly took place during the early implantation period for this group, whereas in the other two groups, it mainly occurred during the later implantation period. These findings align with observed macrophage patterns and osteoconductivity. However, further investigation is needed to determine the correlation between a pro-inflammatory environment and material-associated bone regeneration. Moreover, pro-inflammatory macrophages and multinucleated giant cells demonstrate important role in material degradation. However, the specific advantage of Sr^2+^ and Mg^2+^ on overall osteoregeneration could not be determined in this study. This suggests that the concentration of metal ions utilized in this study may not be optimal for regulating bone remodeling pattern. Consequently, there is an imperative to delve deeper into the immune responses associated with material and tissue regeneration induced by varying metal ion concentrations in the future.

## Data Availability

The original contributions presented in the study are included in the article/Supplementary material, further inquiries can be directed to the corresponding author.
